# Serum Concentration at 24 h With Intensive Beta-Lactam Therapy in Sepsis and Septic Shock: A Prospective Study: Beta-Lactam Blood Levels in Sepsis

**DOI:** 10.1155/2024/9757792

**Published:** 2024-10-21

**Authors:** Evelyne Thériault, Massilia Benali, Samuel Starnino, Hugues Blain, Nicolas Goettel, Bianca Beloin-Jubinville, Amélie Marsot, Francois Lamontagne

**Affiliations:** ^1^Department of Pharmacy, Centre Hospitalier Universitaire de Sherbrooke, Sherbrooke, Québec, Canada; ^2^Faculté de Pharmacie, Université de Montréal, Québec, Canada; ^3^Department of Medicine, Université de Sherbrooke and Centre de Recherche, Centre Hospitalier Universitaire de Sherbrooke, Sherbrooke, Québec, Canada

**Keywords:** beta-lactams, drug monitoring, extended infusion, loading dose, prolonged infusion, sepsis, TDM

## Abstract

**Introduction:** Early administration of appropriate antibiotics has been shown to be among the most effective interventions to reduce mortality in septic patients. We evaluated the attainment of efficacy and safety targets at 24 h associated with the use of intensive beta-lactam therapy in patients admitted to the intensive care unit for sepsis.

**Methods:** This was a prospective study with patients who received beta-lactams for sepsis or septic shock between February 2023 and September 2023. The antibiotic dose was unadjusted for renal function and administered by a loading dose followed by extended infusions, according to local practices. Blood samples were taken at the trough 24 h after the start of the beta-lactam to obtain serum levels. These levels were compared to efficacy and innocuity thresholds found in the literature.

**Results:** Among 36 included patients, all of them achieved serum concentrations above the minimum inhibitory concentration (MIC) for 100% of the therapeutic interval and 75% of them achieved serum concentrations above four times the MIC for 100% of the therapeutic interval. The predefined toxicity thresholds were reached by 8.3% of patients. Renal impairment was the factor most associated with the achievement of higher serum levels.

**Conclusion:** Nonrenally adjusted doses of beta-lactams administered by extended infusion showed good attainment of effective concentrations and few toxic concentrations in critically ill patients with sepsis or septic shock. Further studies are needed to better define the association between toxic concentrations and toxicity manifestations.

## 1. Introduction

Bacterial infections are an important cause of morbidity and mortality in patients admitted to the intensive care unit (ICU). Early administration of an appropriate antibiotic has been shown to be among the most effective interventions to reduce mortality in these patients, with beta-lactams (BLs) being the most frequently prescribed antibiotics in this situation [1–3]. Since BLs are time-dependent antibiotics, their effectiveness depends on the percentage of time that the unbound drug concentration is maintained above the minimum inhibitory concentration (MIC) of the targeted pathogen (expressed as %fT > MIC) [2–4]. It is also known that the extended infusion of BL optimizes the %fT > MIC and reduces the ICU length of stay, duration of mechanical ventilation, and mortality [3, 5].

Critically ill patients are known to have pharmacokinetic (PK) variability secondary to pathophysiological changes such as modifications in the volume of distribution, altered protein binding, or augmented renal clearance [6, 7]. Moreover, estimation of renal function using the serum creatinine level may be biased in critically ill patients [8]. Because of rapid changes in renal function, chronic kidney disease (CKD) formulas should not be used, and urine sample collection is imprecise. The use of BL doses unadjusted for renal function, chronic or acute, has thus been suggested for the first 24 h of treatment, to ensure rapid attainment of therapeutic targets [9, 10].

Different observational studies have used therapeutic drug monitoring (TDM) to evaluate the BL serum levels associated with different dosing regimens in patients with sepsis or septic shock, with the results indicating that about a third of patients do not reach 100% > 1x MIC following administration by either continuous or intermittent infusion [2, 11]. However, the optimal unbound drug concentration target remains uncertain in this setting, with recommendations ranging from one to eight times the MIC of the suspected pathogen. New recommendations suggest a target of 100% fT > 1x MIC to ensure efficacy in the critically ill, while a target of 100% fT > 4x MIC could be used to minimize the development of bacterial resistance and to maximize antibiotic concentration at the infection site [4, 12–15]. Data concerning toxicity thresholds are also variable, with authors suggesting fixed concentrations for different antibiotics [16, 17].

Considering the high morbidity and mortality associated with suboptimal treatment of sepsis and septic shock in the ICU, dosing regimens combining extended infusion and unadjusted renal doses of BL have been suggested, with preoccupations remaining considering the efficacy and safety of this strategy [13, 18]. The aim of this study was to evaluate the use of nonrenally adjusted doses of BL administered by extended infusion in terms of reaching efficacy and safety targets at 24 h using TDM in patients admitted to the ICU for sepsis or septic shock.

## 2. Methods

### 2.1. Study Design and Participants

This observational, prospective study was performed in the three adult ICUs of the Centre Hospitalier Universitaire de Sherbrooke (CHUS). It was funded by the CIUSSS de l'Estrie-CHUS pharmacy department and by the University of Montreal. The trial protocol was approved by the CHUS and University of Montreal's ethics comities. The serum BL assays were performed by the STP^2^ laboratory at the University of Montreal Faculty of Pharmacy.

Included patients were adults aged 18 and older, admitted to the ICU between February 21, 2023, and September 15, 2023, with a confirmed or suspected bacterial infection and receiving piperacillin–tazobactam (PT) or meropenem according to the local BL dosing regimen for patients in the ICU, as described below. Only patients who required vasopressors for their infection were included. Patients were excluded if their BL was changed or stopped within the first 24 h, if they were transferred outside the ICU within 24 h on study BL, or if the studied BL had already been administered for more than 24 h at the time of recruitment. Consent was obtained from the patients or their representatives.

The current practice in the included ICUs consists of administering BL doses nonadjusted for renal function, including acute kidney injury (AKI), CKD, or CRRT, for the first 24 h to rapidly attain therapeutic serum concentrations. The first dose is administered as a bolus over 30 min and subsequent doses as a 3-h infusion, which is referred to as an extended infusion. The subsequent dosing regimen and the treatment duration were determined by the treating team and depended on patient-specific factors and are therefore not presented.

### 2.2. Endpoints

The primary outcome was the achievement of a plasma BL concentration greater than 1x the MIC of the target pathogen for 100% of the therapeutic interval (100% fT > 1x MIC).

The first two secondary outcomes were the achievement of a plasma concentration greater than four times the MIC for 100% of the therapeutic interval (100% fT > 4x MIC) and the achievement of the toxic plasma trough concentration.

The third secondary outcome was the association between achievement of these three targets and clinical impact, assessed by hospital length of stay, duration of mechanical ventilation, time to awakening (time between starting the antibiotic and reaching a score greater than 3 on the Sedation Assessment Scale [SAS] in intubated patients), incidence of delirium (a score greater than 3 on the Intensive Care Delirium Screening Checklist [ICSDC]), incidence of seizures, and mortality at 30 days. The fourth secondary outcome was the assessment of patient characteristics associated with a greater risk of having subtherapeutic or supratherapeutic serum levels.

For the primary and the first two secondary outcomes, we compared the obtained serum concentrations to the MIC of the targeted pathogen. When available, we used the MIC from the antibiogram of the pathogen cultured at the primary site of infection. When no antibiogram was available for an identified pathogen, we used the MIC determined by the Clinical & Laboratory Standards Institute (CLSI) for this specific pathogen [19]. If no pathogen was isolated, we used the highest MIC associated with the most common pathogens found in the identified primary site of infection, according to the Sanford Guide to Antimicrobial Therapy [20]. If no site of infection was identified, we used a MIC of 16 mg/L for PT and 4 mg/L for meropenem, corresponding to the highest MIC expected from susceptible pathogens according to the CLSI [19].

We selected a toxic trough unbound concentration of 157 mg/L for PT and 64 mg/L for meropenem [16, 17, 21].

### 2.3. Post Hoc Analysis

A post hoc analysis was also conducted in which we compared the obtained serum levels to a MIC of 16 mg/L for PT and 4 mg/L for meropenem to allow interpretation of the results in cases where local sensitivities would be less favorable [15].

### 2.4. Blood Samples

Blood samples were taken at the trough, 24 h after starting the studied BL. BL total concentrations were obtained using a validated ultrahigh-performance liquid chromatography method.

Since the bioanalysis method used to measure the serum levels was developed for total concentrations, conversion factors were used to calculate the corresponding unbound concentration of BL. A factor of 70% was chosen for PT [22, 23] and 98% for meropenem [22, 24].

### 2.5. Sample Size

Based on the historical ICU admission data, we estimated a recruitment capacity of 35 to 70 patients for the duration of the study. Using the formula *n*=(*Z*^2^ *p*(1−*P*))/*d*^2^ with an alpha of 0.05 and a proportion of 70% of patients having reached the target of 100% fT > MIC at 24 h as obtained in a trial by De Waele et al. [25], this gives an estimated accuracy of the obtained result between 10% (with 70 patients) and 15% (with 35 patients), which is acceptable for our study.

### 2.6. Statistical Analysis

Statistical analyses were performed by a statistician affiliated with the CHUS. All statistical tests were bilateral, and we considered a significant threshold at 5%. The results were obtained using IBM SPSS Statistics software Version 28. No imputation method was used for missing data.

We obtained dichotomous data for the primary outcome and the first two secondary outcomes. Data are reported with 95% CIs. For the third secondary outcome, we compared dichotomous data of mortality, delirium, and seizures using the Fisher exact test. Continuous variables of hospital length of stay, duration of ventilation, and time to awakening were compared using a Mann–Whitney U test. For the fourth secondary outcome, we calculated relative risks and 95% CIs using the modified Poisson regression with robust error measurement.

## 3. Results

### 3.1. Demographic and Clinical Characteristics

**Figure 1:**
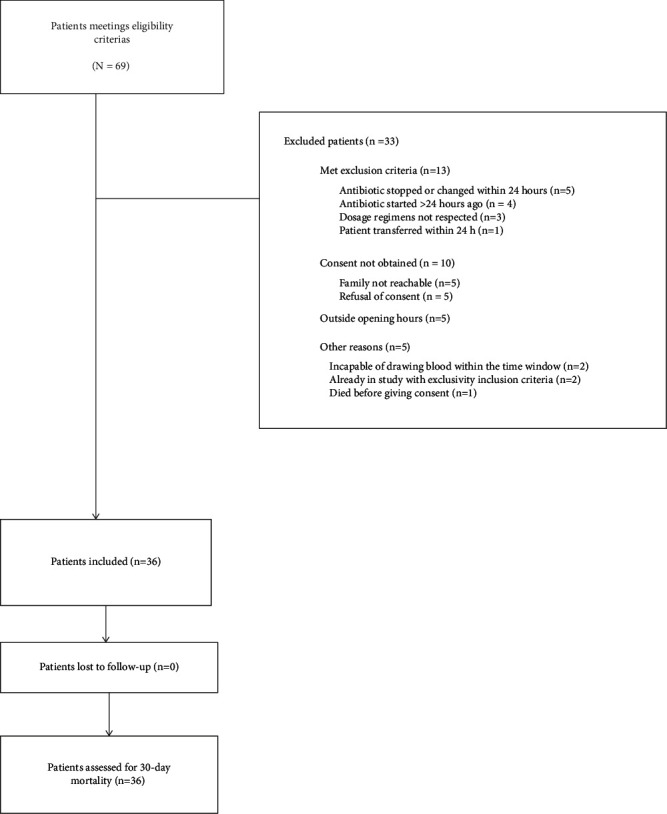
Assessment, exclusions, and follow-up of patients.

Between February 21, 2023, and September 15, 2023, 69 patients were eligible and 36 were included (Figure 1). No patients were lost to follow-up.

Demographic and clinical characteristics are described in Table 1. The mean age was 68.6 years, 27 patients (75%) were men, 29 patients (80.6%) received PT, and 7 (19.4%) received meropenem. The most common infections were intra-abdominal infections (41.7%), urinary tract infections (19.4%), and pulmonary infections (16.7%). Enterobacteriaceae were identified in 38.9% of patients. Eleven patients (30.6%) were mechanically ventilated, 23 patients (69.7%) had CKD (Stages 1–3) [26], 17 patients had AKI (Stages 1–3) [27], and continuous venovenous hemodiafiltration (CVVHDF) was started in the first 24 h in three patients.

For all patients, the administered BL dose was unadjusted for renal function for the first 24 h of treatment. For PT, a dose of 4 g every 6 h was used in 7/29 patients (24%), while a dose of 3 g every 6 h was used in 18/29 patients (62%). In 4/29 patients (14%), the dose was modified between 3 and 4 g every 6 h during the first 24 h of treatment following the clinical decision. For meropenem, clinicians chose a dose of 2 g every 8 h for 2/7 patients (29%) and a dose of 1 g every 8 h for 5/7 patients (71%).

**Figure 2:**
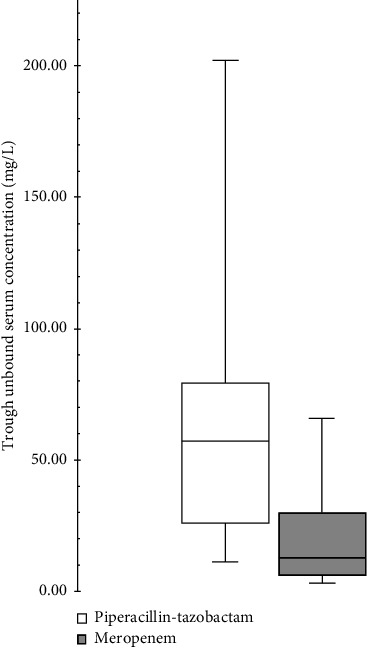
Boxplot of antibiotics through unbound serum concentrations.

As presented in Figure 2, the median (interquartile range) trough unbound serum concentration obtained for PT was 57.2 mg/L (25.8–79.1), while that of meropenem was 13.03 mg/L (5.19–32.63).

### 3.2. Primary Outcome

**Figure 3:**
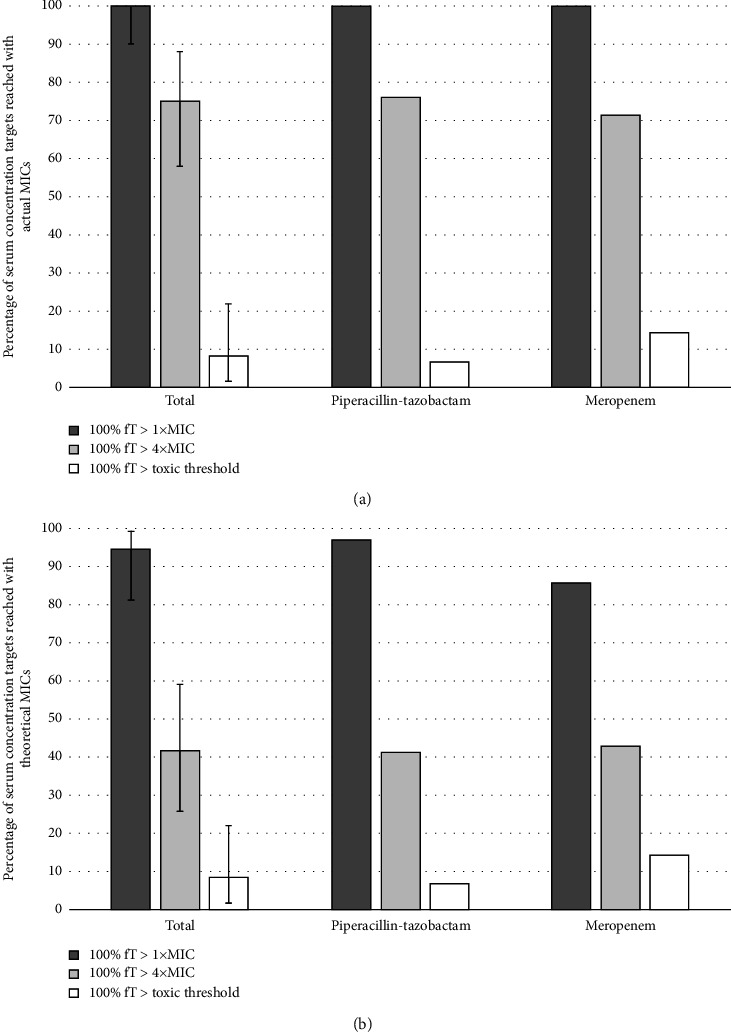
Percentage of serum concentration targets reached (a) with actual MICs and (b) with theoretical MICs.

All patients (36/36) achieved a plasma BL concentration greater than 1x the MIC of the target pathogen for 100% of the therapeutic interval (100% fT > 1x MIC) at 24 h (95% CI: 0.90–1.0) (Figure 3).

### 3.3. Secondary Outcomes

A plasma concentration greater than four times the MIC for 100% of the therapeutic interval (100% fT > 4x MIC) was achieved in 27 patients (75%) (95% CI: 0.58–0.88) (Figure 3). Three patients (8.3%) achieved a toxic plasma trough concentration (95% CI: 0.017–0.22) (Figure 3). Two of them were receiving PT, and one of them was receiving meropenem.

There was no significant difference between patients that achieved 100% fT > 4x MIC and those who did not in terms of clinical impact assessed by mortality at 30 days (*p*=1), hospital length of stay (*p*=0.92), duration of mechanical ventilation (*p*=0.78), time to awakening (*p*=0.39), and incidence of delirium (*p*=0.63) (Table 2).

There was also no significant difference in the outcomes between patients that achieved a toxic plasma through concentration and those who did not (Table 2).

A comparison of patient characteristics of those who did not reach 100% fT > 4x MIC to those who did is detailed in Table 3. There was no significant difference in their age (*p*=0.55), sex (0.22), BMI (*p*=0.86), fluid repletion (*p*=0.66), or fluid balance (*p*=0.46). However, patients with AKI had increased chances of reaching 100% fT > 4x MIC (RR = 1.60 [95% CI: 1.06–2.42], *p*=0.027). The same association was observed with renal replacement therapy (RR = 1.37 [95% CI: 1.12–1.69] *p*=0.003). No significant difference was observed when comparing patients with normal kidney function and patients with all stages of CKD (*p*=0.25). However, it is possible to note that patients with Stage 2 CKD seem to have higher chances of reaching 100% ft > 4x MIC when compared to patients without kidney disease (RR = 1.67 [95% CI: 1.00–2.76] *p*=0.05).

A comparison of patients who reached toxic plasma trough concentration to patients that did not is also detailed in Table 3. These results are based on data from three patients reaching the toxic threshold and their interpretation must only be exploratory.

### 3.4. Post Hoc Analysis

When considering a theoretical MIC of 16 mg/L for PT and 4 mg/L for meropenem, 94.4% of patients (34/36) reached 100% fT > 1x MIC (95% CI: 0.81–0.99) (Figure 3). Of the patients who did not reach 1x MIC, one received PT and the other one meropenem.

A plasma concentration greater than four times the MIC for 100% of the therapeutic interval (100% fT > 4x MIC) was achieved in 15 patients (41.7%) (95% CI: 0.26–0.59) (Figure 3). Among those who did not reach 4x MIC, 17 of them received PT and four of them received meropenem.

There was no significant difference between patients who achieved 100% fT > 1x MIC and those who did not and between patients who achieved 100% fT > 4x MIC and those who did not in terms of clinical impact, as defined above (Table 4).

A comparison of patients who reached 100% fT > 4x MIC to patients who did not is also detailed in the appendix (Table 5). Patients with AKI had higher chances of reaching 100% fT > 4x MIC (RR = 3.67 [95% CI: 1.24–10.85], *p*=0.02). There was no significant difference in all other characteristics.

## 4. Discussion

In sepsis, early administration of an appropriate antibiotic reduces mortality. This prospective study showed that the short-term use of a nonrenally adjusted dose of BL administered by bolus followed by prolonged infusion in the critically ill allowed prompt attainment of efficacity targets in most patients while causing toxic levels in only a few patients.

Considering that the MICs for the pathogens identified for included patients were somewhat low, we added a *post hoc* analysis comparing the obtained serum levels to the CLSI breakpoints for *Pseudomonas aeruginosa*, to allow interpretation of our results in other populations with higher MIC [15, 19, 28].

Our results suggest that the studied dosing regimen allows achievement of 1x MIC in all patients and 4x MIC in most patients, contrary to what is often reported in the literature [2, 29]. A study published in 2014 showed that approximately one-third of patients do not achieve 100% > 1x MIC with PT and meropenem [2]. They also reported that only 30.3% of patients for PT and 41.6% of patients for meropenem reached 4x MIC [2]. The difference in the proportion of patients who achieved 100% > 1x MIC can partially be explained by the fact that 67% of their patients received BL by intermittent infusion. Moreover, they included patients who did not meet criteria for sepsis or septic shock and received BL for prophylaxis indications. It is also important to note that *Pseudomonas aeruginosa* also represented 16% of their identified pathogens but none of ours.

In the *post hoc* analysis using higher theoretical MIC, two patients did not reach 1x MIC. This could indicate that the studied dosing regimen might not be adequate for more resistant pathogens. The proportion of patients reaching 1x MIC is still superior to those reported in the literature, as discussed above [2, 29].

When considering patient characteristics associated with the achievement of 4x MIC, the occurrence of AKI and the use of renal replacement therapy presented a statistically significant association. However, it should be noted that the three patients who underwent CVVHDF were patients who initially presented with significant AKI and had CVVHDF initiated after receiving doses of BL. This association was not observed with CKD as a dichotomous outcome, which was expected considering we mostly recruited patients with Stage 2 and 3 CKD, whereas the monographs recommend adjusting PT doses based on creatinine clearance less than 40 mL/min. However, the large confidence interval highlights that more patients with CKD would have been necessary to better define this association. These results are consequent to the fact that PT and meropenem are mainly renally excreted [23, 30, 31]. Augmented renal clearance was also identified in the literature as a factor associated with lower serum levels [15], but our data did not show any association, with only three patients with creatinine clearance above 130 mL/min.

The number of patients reaching the toxic threshold was too low to draw any strong conclusion on associated outcomes or patient characteristics. These results are also closely linked to the choice of toxic thresholds. It can be noticed that the suggested threshold for PT represents a concentration around 10 times the MIC, while that of meropenem represents a concentration of 16 times the MIC, leaving more room between effective and toxic concentrations.

We can still note that our results are different from what is seen in the literature regarding the proportion of patients exhibiting toxic manifestations following the attainment of toxic thresholds. Authors have reported the occurrence of neurotoxicity in 10%–15% of ICU patients following BL administration and in 50% of patients who reached toxic thresholds [15, 16]. The observed signs of neurotoxicity reported by different authors included confusion, delirium, and seizures, which occurred between 24 h and 30 days following the initiation of the BL [16, 32, 33]. Even though we only collected serum levels after the first 24 h, we did collect data regarding clinical manifestations of neurotoxicity until 24 h after the discontinuation of the studied BL. In our study, no patients had seizures, and the incidence of delirium was not significantly higher in patients with toxic levels, with an overall incidence of 16.7%, which is lower than the expected frequency in the ICU. However, it is important to note that delirium in the ICU is multifactorial and that our study was not powered to assess direct BL contribution to the incidence of delirium [33].

The main risk factor associated with the neurotoxicity of BL is renal impairment [15, 16]. In our study, we can note that all three patients who reached the toxic threshold had severe acute renal impairment, even if we cannot draw any conclusion regarding neurotoxicity manifestations. Our results are relatively reassuring for patients with Stage 1 and 2 AKI, while also highlighting the need to be cautious in patients with anuric Stage 3 AKI. Indeed, although our data demonstrate that four patients with Stage 3 AKI did not reach the toxic thresholds, we were able to note that CVVHDF was started at some point in the first 24 h in three of them. By excluding patients who received renal replacement therapy, a greater proportion of patients with severe AKI, two out of three, presented toxic levels, possibly highlighting a population in which the regimen used may not be as safe as in the rest of the population.

Other trials assessing the BL levels associated with different dosing regimens have used conversions factors representing unbound fraction ranging from 70% to 100% for PT [22, 23, 28, 34, 35]. For the reasons explained above, we chose a factor of 70%, but still wanted to explore our predefined outcomes with the levels obtained with different unbound fractions. No difference was observed in the number of patients reaching toxic threshold when using an unbound fraction of 81% [28] and only one more patient reached a level above 157 mg/L when using an unbound fraction of 90% [34, 35]. These results are reassuring concerning the conversion factor used for our study.

The important variability observed for serum concentrations also highlights the potential usefulness of TDM in assessing efficacity and innocuity, as suggested by different authors [2, 36, 37]. Our study suggests that patients with altered renal function could potentially benefit from TDM to ensure innocuity, but more studies would be needed to evaluate the impact of dose modifications following subtherapeutic or supratherapeutic results [11].

A strength of this study is that it examines both the clinical impacts and the serum levels associated with prolonged infusion of BL, using a validated method to quantify serum concentrations [38]. No patients were lost to follow-up, and we were able to recruit the desired sample size. Our *post hoc* analysis improved external validity, which was, however, impaired by the fact that this study was only conducted in one center. The results of this study are also reassuring with regard to the current clinical practice at the CIUSSS de l'Estrie-CHUS, confirming that our standard regimen allowed the achievement of therapeutic targets in most patients with only a minority of patients reaching toxic thresholds. Along with the recently published international guidelines, this study could encourage other centers to adopt the practice of using extended infusion of B-lactams in patients presenting with sepsis or septic shock. The use of doses unadjusted for renal function for the first 24 h of treatment could also be considered for patients at risk of infection with multidrug-resistant organisms to maximize the drug exposure. Despite the relatively low risk of attaining toxic antibiotic levels at 24 h, access to TDM would be ideal in order to quickly identify patients at risk for toxicity.

This study has some limitations. First, although it was adequate for the chosen study design, the sample size did not allow for subgroup analyses, such as separate analyses for meropenem and PT. Moreover, some results, such as characteristics of patients who reached toxic thresholds, were based on too few patients to be reliable, serving only to generate hypotheses on the subject. We also did not include any patients with initial Stage 4 and 5 CKD, which can have an impact on the number of patients reaching toxic thresholds. However, this represents the real-life population that presented at the CHUS with sepsis and is similar to other trials with septic patients [28].

## 5. Conclusion

Prolonged infusion of a nonadjusted dose of BL for the first 24 h of treatment in patients admitted to the ICU for sepsis or septic shock was associated with attainment of targets of 1x MIC in all patients and of 4x MIC in 75% of patients, with only 8.3% of patients reaching theoretical toxic levels after 24 h, suggesting that this regimen is safe and effective. Uncertainty remains concerning the best way to maintain adequate levels for the rest of the treatment course. No differences in clinical outcomes were observed between the different groups of target attainment. Altered renal function was the most associated factor with the achievement of higher serum levels.

## Figures and Tables

**Table 1:** Demographic and clinical characteristics.

**Characteristics**	**N = 36**
Age (years)—mean ± *σ*	68.6 ± 16.8
Male sex—*n* (%)	27 (75)
BMI (calculated) (kg/m^2^)—mean ± *σ*	27.5 ± 6.8
Serum albumin (g/L)—mean ± *σ*	22.1 ± 4.6 (*n* = 12/36)
Chronic kidney disease—*n* (%)	
Normal kidney function	10/33 (30.3)
Stage 1	15/33 (45.5)
Stage 2	4/33(12.1)
Stage 3	4/33 (12.1)
Stages 4–5	0/33
Acute kidney injury—*n* (%)	
No AKI	17/34 (50.0)
Stage 1	7/34 (20.6)
Stage 2	4/34 (11.8)
Stage 3	6/34 (17.6)
APACHE II score—mean ± *σ*	23.2 ± 12.1
Antibiotic—*n* (%)	
Piperacillin–tazobactam^†^	29 (80.6)
Meropenem^†^	7 (19.4)
Infection sites—*n* (%)	
Intra-abdominal	15 (41.7)
Urinary	7 (19.4)
Pulmonary	6 (16.7)
Osteomyelitis/necrotizing fasciitis	3 (8.3)
Soft tissue	2 (5.6)
Cardiac	2 (5.6)
Effusion/empyema	1 (2.8)
Catheters	1 (2.8)
Not identified	2 (5.6)
Isolated bacteria—*n* (%)	
*Enterobacteriaceae*	14 (38.9)
*Streptococcus*	6 (16.7)
*Anaerobes*	5 (13.9)
*Staphylococcus*	2 (5.6)
*Enterococcus*	1 (2.8)
H. influenzae	1 (2.8)
Not isolated	14 (38.9)
MIC of identified pathogens (mg/L)—median (interquartile range)	
Piperacillin–tazobactam	4 (1–8)
Meropenem	1 (0.375–3)
Comedication^‡^-*n* (%)	
Other antibiotics	12 (33.3)
Fluid repletion (mL)^‡^—mean ± *σ*	6322 ± 2720
Fluid balance (mL)^‡^—mean ± *σ*	4333 ± 2553
Renal replacement therapy^†^—*n* (%)	
Hemodialysis	0
CVVHDF	3 (8.3)
Mechanical ventilation—*n* (%)	11 (30.6)
Severity of illness^§^	
Sepsis	16/35 (45.7)
Septic shock	19/35 (54.3)

^†^PT, meropenem, and imipenem were the three BLs administered by prolonged infusion at the CIUSSSE-CHUS ICU. However, no patients meeting the inclusion criteria received imipenem during the study period.

^‡^From diagnosis to first BL sampling.

^§^According to sepsis-4 criteria.

**Table 2:** Association between target achievement and clinical impact.

**4 x MIC**	**100% fT < 4 x MIC (n = 9)**	**100% fT ≥ 4 x MIC (n = 27)**	**p value**
Mortality at 30 days—*n* (%)	1 (11.1)	4 (14.8)	1.00
Delirium—*n* (%)	2 (22.2)	4 (14.8)	0.63
Seizures—*n* (%)	0	0	-
Hospital length of stay (days)—mean ± *σ*	20.1 ± 24.1	16.9 ± 19	0.92
Duration of mechanical ventilation (days)—mean ± *σ*	2.48 ± 2.23	3.89 ± 5.27	0.78
Time to awakening (hours)—mean ± *σ*	43.8 ± 26.5	22.9 ± 23.7	0.39

**Toxic threshold**	**Toxic trough levels (n = 3)**	**Nontoxic trough levels (n = 33)**	**p value**

Mortality at 30 days—*n* (%)	1 (33.3)	4 (12.1)	0.37
Delirium—*n* (%)	1 (33.3)	5 (15.1)	0.43
Seizures—*n* (%)	0	0	—
Hospital length of stay (days)—mean ± *σ*	29.3 ± 36.1	16.9 ± 19.4	0.33
Duration of ventilation (days)—mean ± *σ*	0.92 ± 0.45	4.08 ± 5.0	0.58
Time to awakening (hours)—mean ± *σ*	15.8 ± NA	32.9 ± 27.1	0.75

**Table 3:** Comparison of patient characteristics who had subtherapeutic or supratherapeutic serum levels to those who had reached target levels.

**Characteristics**	**100% fT ≥ 4x MIC not reached (n = 9)**	**100% fT ≥ 4x MIC reached (n = 27)**	**RR [95% CI]**	**p value**
Age (years)—mean ± *σ*	66.0 ± 15.9	69.5 ± 17.1	1.00 [0.99–1.02]	0.55
Male sex—*n*. (%)	5 (55.6)	22 (81.5)	1.47 [0.80–2.70]	0.22
BMI (kg/m^2^)—mean ± *σ*	27.2 ± 5.7	27.6 ± 7.29	1.00 [0.97–1.03]	0.86
Fluid repletion (mL)^†^—mean ± *σ*	6667 ± 3564	6207 ± 2468	1.00 [1.00–1.00]	0.66
Fluid balance (mL)^†^—mean ± *σ*	4887 ± 3332	4148 ± 2325	1.00 [1.00–1.00]	0.46
Chronic kidney disease—*n*. (%)				
Normal kidney function	4/8 (50)	6/25 (24)		
Stage 1	3/8 (37.5)	12/25 (48)	1.33 [0.76–2.35]	0.32
Stage 2	1/8(12.5)	4/25 (16)	1.67 [1.00–2.76]	0.05
Stage 3	0/8	3/25 (12)	1.25 [0.58–2.67]	0.56
Missing data	1 (11)	2 (7)	—	—
Acute kidney injury—*n* (%)				
No AKI	7/8 (87.5)	10/26 (38.5)		
Stage 1	0/8	7/26 (26.9)	1.70 [1.14–2.53]	0.009
Stage 2	1/8 (12.5)	3/26 (11.5)	1.27 [0.64–2.55]	0.49
Stage 3	0/8	6/26 (23.1)	1.70 [1.14–2.53]	0.009
Missing data	1 (11)	1 (4)		
Renal replacement therapy^†^—*n* (%)	0	3 (11.1)	1.37 [1.12–1.69]	0.003
Antibiotic—*n* (%)				
Piperacillin–tazobactam	7 (77.8)	22 (81.5)		
Meropenem	2 (22.2)	5 (18.5)		

**Characteristics**	**Toxic trough levels (n = 3)**	**Nontoxic trough levels (n = 33)**	**R** **R** [95% **CI**]^‡^	**p value** ^‡^

Age (years)—mean ± *σ*	77 ± 6.67	67.8 ± 17.5	—	—
Male sex—*n* (%)	2 (66.7)	25 (75.8)	—	—
BMI (kg/m^2^)—mean ± *σ*	26.1 ± 2.2	27.6 ± 7.14	—	—
Fluid repletion (mL)^†^—mean ± *σ*	8704 ± 2802	6105 ± 2612	—	—
Fluid balance (mL)^†^—mean ± *σ*	7828 ± 3099	4015 ± 2263	—	—
Chronic kidney disease—*n* (%)				
Normal kidney function	0	10/30 (33.3)	—	—
Stage 1	2 (66.7)	13/30 (43.3)	—	—
Stage 2	1 (33.3)	3/30 (10)	—	—
Stage 3	0	4/30 (13.3)	—	—
Missing data	0	3 (9)	—	—
Acute kidney injury—*n* (%)				
No AKI	0	17/31 (54.8)	—	—
Stage 1	0	7/31 (22.6)	—	—
Stage 2	1 (33.3)	3/31 (9.7)	—	—
Stage 3	2 (66.7)	4/31 (12.9)	—	—
Missing data		2 (6)	—	—
Renal replacement therapy^†^—*n* (%)	0	3 (9.1)	—	—
Antibiotic—*n* (%)				
Piperacillin–tazobactam	2 (66.7)	27 (81.8)	—	—
Meropenem	1 (33.3)	6 (18.2)	—	-

^†^From diagnosis to first BL sampling.

^‡^Because of the small number of patients with toxic trough levels, accurate RR and *p* value could not be extracted.

**Table 4:** Association between targets achievement and clinical impact—post hoc analysis.

**1x MIC**	**100% fT ≥ MIC reached (n = 34)**	**100% fT ≥ MIC not reached (n = 2)**	**p value** ^†^
Mortality at 30 days—nb. (%)	5 (14.7)	0	—
Delirium—nb. (%)	6 (17.6)	0	—
Seizures—nb. (%)	0	0	—
Hospital length of stay (days)—mean ± *σ*	17.7 ± 20	18.6 (30.2)	—
Duration of mechanical ventilation (days)—mean ± *σ*	1.81 ± 4.39	0	—
Time to awakening (hours)—mean ± *σ*	23.05 ± 22.06	0	—

	**100% fT < 4 x MIC (n = 21)**	**100% fT ≥ 4 x MIC (n = 15)**	**p value**

Mortality at 30 days—*n* (%)	2 (9.5)	3 (20)	0.63
Delirium—*n* (%)	3 (14.3)	3 (20)	0.68
Seizures—*n* (%)	0	0	**—**
Hospital length of stay (days)—mean ± *σ*	17.1 ± 18.2	18.5 ± 23.6	0.76
Duration of mechanical ventilation (days)—mean ± *σ*	2.09 ± 1.87	4.69 ± 6.31	0.93
Time to awakening (hours)—mean ± *σ*	34.6 ± 31.6	26.9 ± 26	0.89

^†^Because of the small number of patients with 100% fT >MIC not reached, accurate p value could not be extracted.

**Table 5:** Comparison of patient characteristics who had subtherapeutic or supratherapeutic serum levels to those who had reached target levels—post hoc analysis.

**Characteristics**	**100% fT ≥ 4x MIC not reached (n = 21)**	**100% fT ≥ 4x MIC reached (n = 15)**	**RR [95% CI]**	**p value**
Age (years)—mean ± *σ*	67.3 ± 18.3	70.5 ± 14.8	1.00 [0.97–1.04]	0.57
Male sex—*n*. (%)	14 (66.7)	13 (86.7)	2.17 [0.60–7.82]	0.24
BMI (kg/m^2^)—mean ± *σ*	28.00 ± 6.99	26.6 ± 6.81	0.97 [0.91–1.05]	0.50
Fluid repletion (mL)^†^—mean ± *σ*	6452 ± 2790	6140 ± 2729	1.00 [1.00–1.00]	0.69
Fluid balance^†^ (mL)—mean ± *σ*	4229 ± 2404	4478 ± 2883	1.00 [1.00–1.00]	0.74
Chronic kidney disease—*n*. (%)				
Normal kidney function	9/20 (45)	1/13 (7.7.)	—	—
Stage 1	10/20 (50)	5/13 (38.5)	3.33 [0.46–24.44]	0.24
Stage 2	0/20	4/13 (30.8)	10.00 [1.56–64.2]	0.02
Stage 3	1/20 (5)	3/13(23.1)	7.50 [1.07–52.38]	0.04
Missing data	1 (5)	2 (13)	—	—
Acute kidney injury—*n* (%)				
No AKI	14/20 (70)	3/14 (21.4)	—	—
Stage 1	4/20 (20)	3/14 (21.4)	2.43 [0.64–9.24]	0.19
Stage 2	1/20 (5)	3/14 (21.4)	4.25 [1.32–13.73]	0.02
Stage 3	1/20 (5)	5/14 (35.7)	4.72 [1.59–14.01]	0.005
Missing data	1 (5)	1 (7)	—	—
Renal replacement therapy^†^—*n* (%)	1 (4.8)	2 (13.3)	1.69 [0.68–4.18]	0.25
Antibiotic—*n* (%)				
Piperacillin–tazobactam	17 (81)	12 (80)	—	—
Meropenem	4 (19)	3 (20)	—	—

^†^From diagnosis to first BL sampling.

## Data Availability

The data that support the findings of this study are available from the corresponding author, Theriault E, upon reasonable request.
